# Relationships between plasma fatty acids in adults with mild, moderate, or severe COVID-19 and the development of post-acute sequelae

**DOI:** 10.3389/fnut.2022.960409

**Published:** 2022-09-14

**Authors:** Sophia Stromberg, Bridget A. Baxter, Gregory Dooley, Stephanie M. LaVergne, Emily Gallichotte, Taru Dutt, Madison Tipton, Kailey Berry, Jared Haberman, Nicole Natter, Tracy L. Webb, Kim McFann, Marcela Henao-Tamayo, Greg Ebel, Sangeeta Rao, Julie Dunn, Elizabeth P. Ryan

**Affiliations:** ^1^Department of Food Science and Human Nutrition, Colorado State University, Fort Collins, CO, United States; ^2^Department of Environmental and Radiological Health Sciences, Colorado State University, Fort Collins, CO, United States; ^3^Department of Microbiology, Immunology and Pathology, Colorado State University, Fort Collins, CO, United States; ^4^Department of Clinical Sciences, Colorado State University, Fort Collins, CO, United States; ^5^University of Colorado Health, Medical Center of the Rockies, Loveland, CO, United States

**Keywords:** COVID-19, plasma fatty acid profiles, persistent symptoms, disease severity, post-acute sequelae of COVID-19 (PASC), nutritional status, SARS-CoV-2

## Abstract

**Background:**

SARS-CoV-2 has infected millions across the globe. Many individuals are left with persistent symptoms, termed post-acute sequelae of COVID-19 (PASC), for months after infection. Hyperinflammation in the acute and convalescent stages has emerged as a risk factor for poor disease outcomes, and this may be exacerbated by dietary inadequacies. Specifically, fatty acids are powerful inflammatory mediators and may have a significant role in COVID-19 disease modulation.

**Objective:**

The major objective of this project was to pilot an investigation of plasma fatty acid (PFA) levels in adults with COVID-19 and to evaluate associations with disease severity and PASC.

**Methods and procedures:**

Plasma from adults with (*N* = 41) and without (*N* = 9) COVID-19 was analyzed by gas chromatography-mass spectrometry (GC-MS) to assess differences between the concentrations of 18 PFA during acute infection (≤14 days post-PCR + diagnosis) in adults with varying disease severity. Participants were grouped based on mild, moderate, and severe disease, alongside the presence of PASC, a condition identified in patients who were followed beyond acute-stage infection (*N* = 23).

**Results:**

Significant differences in PFA profiles were observed between individuals who experienced moderate or severe disease compared to those with mild infection or no history of infection. Palmitic acid, a saturated fat, was elevated in adults with severe disease (*p* = 0.04), while behenic (*p* = 0.03) and lignoceric acid (*p* = 0.009) were lower in adults with moderate disease. Lower levels of the unsaturated fatty acids, γ-linolenic acid (GLA) (*p* = 0.03), linoleic (*p* = 0.03), and eicosapentaenoic acid (EPA) (*p* = 0.007), were observed in adults with moderate disease. Oleic acid distinguished adults with moderate disease from severe disease (*p* = 0.04), and this difference was independent of BMI. Early recovery-stage depletion of GLA (*p* = 0.02) and EPA (*p* = 0.0003) was associated with the development of PASC.

**Conclusion:**

Pilot findings from this study support the significance of PFA profile alterations during COVID-19 infection and are molecular targets for follow-up attention in larger cohorts. Fatty acids are practical, affordable nutritional targets and may be beneficial for modifying the course of disease after a COVID-19 diagnosis. Moreover, these findings can be particularly important for overweight and obese adults with altered PFA profiles and at higher risk for PASC.

**Clinical trial registration:**

[ClinicalTrials.gov], identifier [NCT04603677].

## Introduction

SARS-CoV-2, the virus that causes COVID-19, has rapidly spread across the world, killing over 6 million people worldwide since its initial identification in December 2019 (1). Many have speculated that the high prevalence of metabolic dysfunction, obesity, and chronic disease in the United States (US) have contributed to morbidity and mortality surrounding COVID-19 (2, 3). Emerging evidence shows that obesity is an important risk factor for COVID-19 hospitalization and need for supplemental oxygen as well as poor disease outcomes and development of post-acute sequelae of COVID-19 (PASC) (4, 5).

The definition of PASC by the World Health Organization (WHO) describes symptoms that begin during acute COVID-19 infection (or shortly after) and last for at least two months following diagnosis without any alternative explanation (6). Common symptoms of PASC include fatigue, shortness of breath, and cognitive dysfunction, though there is notable breadth and diversity in the reporting of symptoms by those who experience and are diagnosed with this condition (4, 6). Females and individuals of more advanced age have been found to report a higher incidence of lasting symptoms, with the most common being extreme fatigue, headaches, dyspnea, and persistent anosmia (7). The percentage of individuals who experience PASC varies between countries and cohorts, though some have indicated over 50% of patients may experience lasting physical, emotional, or mental deficits (8–11).

Although the mechanisms causing severe COVID-19 are not definitively known, there has been some evidence to suggest that higher levels of inflammation may contribute to severe disease during acute infection and PASC (8, 9, 12, 13). Obesity is associated with higher levels of pro-inflammatory mediators, which may predispose individuals to a dysregulated immune response (14). Excess adipose tissue is a major source of the pro-inflammatory mediators observed in obesity, and this hyperinflammatory state can be further exacerbated by dietary inadequacies (15). Fatty acids are key modulators of inflammatory pathways within the body, and a high intake of saturated fatty acids with low intake of unsaturated fatty acids has been linked to a greater degree of systemic inflammation (16). Indeed, previous studies have found dyslipidemia and altered free fatty acid metabolism to be associated with COVID-19 infection (17–20). Additional studies have also shown that inflammatory downstream metabolites of certain fatty acids may contribute to increased pulmonary inflammation and vascular permeability, leading to a greater likelihood of developing acute respiratory distress syndrome (ARDS) during COVID-19 infection (21, 22). Thus, individuals with unfavorable fatty acid profiles may be at heightened risk for a more severe COVID-19 disease course.

This present study explores relationships between systemic fatty acid profiles and COVID-19 disease severity as well as in relation to the development of PASC.

## Materials and methods

### Participant identification

This study is part of the Northern Colorado Coronavirus Biobank (NoCO CoBIO): a biorepository for acute and convalescent patients. Colorado State University (CSU) and University of Colorado Health (UCHealth) networks were used to recruit COVID-19 survivors to participate in an observational, longitudinal cohort study, as previously described (5, 23). Inclusion criteria for this study were a positive SARS-CoV-2 polymerase chain reaction test (PCR). Individuals who received a COVID-19 diagnosis *via* home test kits or antigen tests were not included. Participants were also required to be at least 18 years of age. Exclusion criteria included pregnancy or incarceration at time of enrollment. Participants consented to undergo four study clinic visits: at enrollment and approximately 1, 4, and 6 months after enrollment with the choice for a one year follow-up. The complete account of all recruitment, enrollment, and data acquisition procedures and rationale was previously described (23). The subset of 50 participants included in this present study completed three study visits, deemed visit 1 (V1), visit 2 (V2), and visit 3 (V3). Study visit 1 was at time of enrollment, which was either during acute-stage infection (≤14 days post PCR+) or during the convalescent stage (≥14 days post PCR+). Study visit 2 was approximately 30 days after V1, and V3 was approximately 90 days after V1. The Northern Colorado Coronavirus Biobank has been approved by CSU’s Institutional Review Board [IRB; protocol 2105 (20-10063H)] and UCHealth IRB (Colorado Multiple IRB 20-6043) and is registered at ClinicalTrials.gov (NCT04603677). All participants provided written informed consent. The biorepository was in accordance with the Helsinki Declaration and its 2013 amendments. A convenience sample of uninfected adults with no history of COVID-19 infection and with a negative SARS-CoV-2 PCR test were enrolled for the same study visit and specimen collections. 140 diagnosed and 18 uninfected adults completed their study visits between July 2020 and March 2021. Twenty-five-dollar cash compensation was given to all participants at each study visit.

### Group stratification and clinical data acquisition

Participants were categorized as having mild, moderate, or severe disease based on the Yale Impact Scoring during their acute stage of infection, which is defined as the first 14 days of infection following a positive SARS-CoV-2 PCR test (24). Individuals who required greater than 5 L of supplemental oxygen were classified as having severe disease, while those who were hospitalized but required 1–5 L had moderate disease, and no oxygen requirement was classified as mild disease. Individuals who did not have a documented history of COVID-19 were also included in this study as uninfected. To assess for risk factors of disease severity, clinical data, including age, body mass index (BMI), comorbidities, and race/ethnicity, were collected on each participant either at clinic visits (*N* = 19) or obtained by hospital electronic medical records (*N* = 22). All demographic data from adults without COVID-19 infection was obtained at clinic visits (*N* = 9). This information was de-identified and stored in the password-protected Research Electronic Data Capture (REDCap) web platform. Participants provided written informed consent to a scheduled series of longitudinal visits following the PCR confirmed diagnosis, at which time a 70-symptom survey was administered. Results from this survey were used to identify new or persistent PASC. Participants were interviewed by clinic staff and asked to identify whether any previously reported symptoms were continuing. Participants were defined as having PASC according to the WHO guidelines, which defines this condition by the persistence of at least one of the following symptoms: fatigue, dyspnea, joint pain, chest pain, confusion, difficulty concentrating, forgetfulness or absent-mindedness for at least 60 days post acute infection (6). Dyspnea was defined as difficulty breathing or shortness of breath in participants.

### Fatty acid profiling

The quantification of lauric, myristate, pentadecanoic, palmitic, palmitoleic, steric, oleic, linoleic, γ_linolenic, linolenic, arachidic, cis_11_Eicosenoic, arachidonic, cis_5_8_11_14_17_eicosapentaenoic, behenic, cis_13_16_ docosadienoic, cis_4_7_10_13_16_19_Docosahexaenoic, and lignoceric acids were conducted on participant plasma samples according to standardized methods defined by the National Institute of Health (25). 100 μl of plasma, 10 μl of non-adecanoic acid (1 μg/ml) as an internal standard, and 1.5 ml of methanol were added to glass test tube, and the solution was vortexed. 0.1 ml of acetyl chloride was added while lightly vortexing the sample to mix. The capped samples were then derivatized at 90°C/min for 45 min. Following the incubation period, the samples were allowed to come to room temperature prior to adding 1.5 ml 6% sodium bicarbonate and 0.5 ml hexane. The samples were vortexed for approximately one minute and then placed in the centrifuge at 3,220 × *g* for 5 min. The upper hexane layer containing fatty acid methyl esters (FAME) was transferred in the autosampler vial for analysis by gas chromatography-mass spectrometry.

Samples were analyzed with an Agilent 6890 Gas Chromatograph and a Micromass Quattro Micro Mass Spectrometer. FAMEs were analyzed with a 1 μl sample injection and a 100:1 split ratio onto a Restek FAMEWAX column (30 m × 0.25 mm × 0.25 μm). The oven temperature profile was 50°C for 1 min to 200°C at 25°C/min, to 230°C at 3°C/min, then held at 230°C for 33 min. The flow rate of helium was 1 ml/min, inlet temperature was 275°C, and the GC-mass spectrometer interface temperature was set at 280°C. The mass spectrometer was operated in selected ion monitoring (SIM) mode for the fragment ions of 55, 67, 69, 74, 79, 81, 87, 91, 95, and 99 m/z. The data collection and processing were performed by using Waters™ MassLynx software. Quantitation was performed with linear regression using 7-point calibration curves from 30 to 600 μg/ml.

### Statistical analysis

The fatty acid data were continuous hence was evaluated for normality assumption prior to performing a linear mixed model. If the data were not normal, it was converted into log scale before performing the analysis. A linear mixed model was performed to compare the fatty acids between disease severity categories at baseline and between participants that developed PASC versus participants without PASC. Fatty acid profiles at 37.2 ± 33.2 days post-PCR + diagnosis were compared to those obtained 72.5 ± 34.8 and 134.0 ± 38.9 days post-PCR + diagnosis, with PASC vs No PASC, and interaction effects included as fixed effects in the mixed model. Tukey’s method was used to adjust for multiple comparisons, and BMI was included as a covariate for analysis. A *p*-value of 0.05 was used as criteria to determine statistical significance. SAS v9.4 (SAS Institute Inc., Cary, NC, United States) was used to perform all statistical analyses. GraphPad Prism version 9 was used for all figures.

## Results

### Study cohort

Fifty participants were evaluated for fatty acid profiles (Table 1). Twenty participants diagnosed as having a mild acute infection (required no oxygen) had a mean age of 38 years and a mean BMI of 23.6. Seventy-five percent were women, and 15% were hospitalized. Twelve adults experienced a moderate infection (required 1–5 L oxygen) with a mean age of 57.1 years and a mean BMI of 35.5; fifty-eight percent were women, 42% received convalescent plasma, and 83% were hospitalized. Nine participants experienced a severe infection (>5L oxygen) with a mean age of 55.8 years and mean BMI of 39.7. Forty-five percent of the participants with severe disease were women, 89% received convalescent plasma, and 100% were hospitalized. Additionally, nine uninfected (no COVID-19 diagnosis) participants were enrolled with a mean age of 50 years and mean BMI of 23.2. 78% of the uninfected individuals were female.

**TABLE 1:** Baseline adult participant characteristics by COVID-19 disease severity (*N* = 50).

Characteristics	Uninfected (*N* = 9)	Mild (*N* = 20)	Moderate (*N* = 12)	Severe (*N* = 9)
Age, mean + SD, year	50 + 9.3	38.1 + 18.2	56.8 ± 14.9	55.8 ± 13.1
**Sex, no. (%)**				
Female	7 (78)	15 (75)	7 (58)	4 (45)
Male	2 (22)	5 (25)	5 (42)	5 (56)
BMI, mean ± SD	23.2 + 3.1	23.6 + 5.8	35.5 + 10.8	39.7 + 11.7
**Ethnicity, no. (%)**				
Non-Hispanic/Latinx	9 (100)	19 (95)	10 (83)	6 (67)
Hispanic/Latinx	0 (0)	1 (5)	2 (17)	3 (33)
Hospitalized	–	3 (15)	10 (83)	9 (100)
Non-hospitalized	–	17 (85)	2 (17)	0 (0)
Convalescent Plasma	–	1 (5)	5 (42)	8 (89)
**Pre-existing conditions no. (%)**				
COPD	0 (0)	1 (5)	3 (25)	3 (33)
DM	0 (0)	2 (10)	5 (42)	6 (67)
HTN	0 (0)	1 (5)	5 (42)	8 (89)
CAD	0 (0)	1 (5)	1 (8)	1 (11)
Asthma	0 (0)	0 (0)	3 (25)	1 (11)
Liver disease (unspecified)	0 (0)	0 (0)	1 (8)	1 (11)

BMI denotes body mass index; Pre-existing conditions were self-reported for non-hospitalized participants and retrieved from electronic medical records and self-reported for hospitalized participants. COPD, chronic obstructive pulmonary disease; DM, diabetes mellitus; HTN, hypertension; CAD, coronary artery disease.

### Saturated fatty acids

**FIGURE 1:**
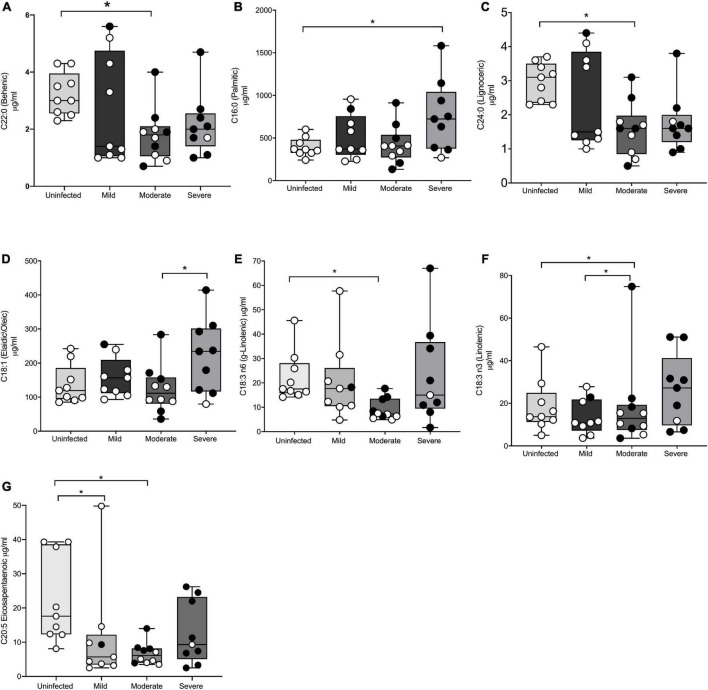
Significant differences in fatty acid profiles between disease severity groups and the uninfected adults. **(A)** Behenic acid lower in moderate adults compared to uninfected. **(B)** Palmitic acid elevated in adults with severe disease compared to uninfected. **(C,E)** Lignoceric acid and γ-linolenic acid lower in moderate disease compared to uninfected. **(D)** Oleic acid lower in moderate disease compared to severe disease. **(F)** Linolenic acid lower in moderate adults compared to mild disease and uninfected. **(G)** EPA lower in mild and moderate adults compared to uninfected. Shaded points represent adults who received convalescent plasma. * denotes statistical significance *p* ≤ 0.05.

Thirty-seven adults (9 mild, 10 moderate, 9 severe, and 9 uninfected) were analyzed for baseline fatty acids levels in plasma at V1. Table 2 shows seven saturated fatty acids from plasma were quantified and analyzed for significance in disease severity during acute infection (≤14 days post-PCR + diagnosis). Figure 1A, shows mean behenic acid (C22) was significantly lower in the adults with moderate disease when compared to the uninfected adults (1.8 vs 3.2 μg/ml, *p* = 0.03). There was no significant difference in behenic acid levels in adults with mild disease or severe disease compared to adults with no prior history of infection (*p* = 0.36 and *p* = 0.22, respectively). Palmitic acid (C16) was significantly higher in the infected adults with severe disease compared to the uninfected adults (755.1 vs 513.3 μg/ml, *p* = 0.04) (Table 2; Figure 1B). Those in the mild and moderate disease severity groups did not show significantly different levels in palmitic acid compared to the uninfected (*p* = 0.79 and *p* = 0.99, respectively). Levels of lignoceric acid (C23) were significantly lower in the moderate infected adults compared to the uninfected adults (1.6 vs 2.9 μg/ml, *p* = 0.009) (Table 2; Figure 1C). Lauric (C12), stearic (C18), pentadecanoic (C15), and arachidic acid (C20) levels were not significantly different between the different disease severity groups or compared to the uninfected adults (Table 2).

**TABLE 2:** Baseline fatty acid profile in plasma for mild, moderate, and severe COVID-19 disease severity compared to uninfected adults.

	Uninfected (*N* = 9)	Mild (*N* = 20)	*p*-value			Moderate (*N* = 12)	*p*-value		Severe (*N* = 9)	*p*-value
			Mild moderate	Mild severe	Mild uninfected		Moderate severe	Moderate uninfected		Severe uninfected
**Saturated fatty acids (μg/ml)**										
Behenic (C22)	3.2 ± 0.7	2.7 ± 1.9	0.67	0.99	0.36	1.8 ± 0.9	0.84	**0.03**	2.2 ± 1.1	0.22
Palmitic (C16)	393.4 ± 109.5	513.4 ± 262.3	0.9	0.26	0.79	427.5 ± 224.7	0.07	0.99	755.1 ± 419.8	**0.04**
Lignoceric (C23)	2.9 ± 0.5	2.4 ± 1.4	0.27	0.71	0.43	1.6 ± 0.8	0.811	**0.009**	1.8 ± 0.9	0.06
Lauric (C12)	3.5 ± 3.3	3.2 ± 2.1	0.97	0.92	1	3.8 ± 2.6	0.99	0.96	7.0 ± 13.0	0.90
Stearic (C18)	178.0 ± 28.2	180.4 ± 73.5	0.39	0.99	0.99	129.9 ± 64.1	0.3	0.44	186.5 ± 94.6	0.99
Pentadecaoic (C15)	3.7 ± 1.9	3.9 ± 3.1	0.97	0.77	0.98	3.1 ± 2.8	0.49	0.83	5.0 ± 3.9	0.94
Arachidic (C20)	1.4 ± 0.3	1.4 ± 0.8	0.92	0.99	0.94	1.1 ± 0.5	0.76	0.61	1.4 ± 0.8	0.99
**Monounsaturated fatty acids (μg/ml)**										
Oleic (C18:1n9)	137.3 ± 57.2	158.8 ± 57.4	0.43	0.66	0.86	123.8 ± 69.9	**0.04**	0.85	220.1 ± 108.9	0.27
Myristoleic (C14:1n5)	0.7 ± 0.3	0.8 ± 0.4	1	0.99	0.97	0.8 ± 0.4	0.99	0.98	1.0 ± 1.3	0.91
Eicosenoic (C20:1n9)	1.9 ± 0.9	3 ± 1.7	0.69	0.7	0.46	2.2 ± 1.5	0.14	0.97	3.8 ± 1.9	0.07
Palmitoleic (C16:1n7)	24.3 ± 12.7	46.4 ± 34.3	0.99	0.56	0.63	41.0 ± 17.4	0.72	0.43	70.3 ± 59.2	0.08
**Polyunsaturated fatty acids (μg/ml)**										
γ-linolenic (C18:3n6)	22.4 ± 10.2	20.4 ± 15.9	0.18	0.99	0.87	9.1 ± 4.4	0.24	**0.03**	23.2 ± 20.4	0.8
Linoleic (C18:2n9)	536.2 ± 83.3	527.9 ± 222.9	**0.04**	0.8	0.99	305.8 ± 175.8	0.29	**0.03**	452.6 ± 196.2	0.75
Eicosapentaenoic (C20:5n3)	22.4 ± 12.8	11.4 ± 14.9	0.96	0.84	**0.03**	6.6 ± 3.2	0.53	**0.007**	12.6 ± 9.2	0.18
Docasahexaenoic (C22:6n3)	45.7 ± 18.6	39.7 ± 30.6	0.99	0.72	0.77	47.1 ± 26.8	0.83	0.87	50.3 ± 32.7	0.99
Arachidonic (C20:4n6)	205.2 ± 39.2	199.3 ± 104.2	1	0.84	0.91	189.3 ± 79.5	0.81	0.89	241.8 ± 147.2	0.99
Linolenic (C18:3n3)	18.6 ± 12.5	13.6 ± 8.3	0.99	0.31	0.8	18.3 ± 20.7	0.47	0.93	26.3 ± 16.9	0.83

Values presented as mean ± standard deviation. Linear mixed model was performed to compare the fatty acids between disease severity categories and the uninfected adults. SAS v9.4 (SAS Institute Inc., Cary, NC, United States) was used to perform all statistical analyses. P < 0.05 significant; bold = significant.

### Monounsaturated fatty acids

A total of four monounsaturated fatty acids were quantified and analyzed for significant differences between the disease severity groups and the uninfected at baseline (Table 2). Figure 1D shows oleic acid (C18:1n9) was significantly different between the moderate infected adults (123.8 μg/ml) and severe infected adults (220.1 μg/ml), with those in the moderate disease group demonstrating a lower level of this fatty acid compared to the severe group (*p* = 0.04). The mean BMI of the moderate and severe disease groups was similar (35.5 vs 39.7 kg/m^2^, respectively), thus indicating the significance of this finding was independent of BMI. There was no significant difference in oleic acid levels observed in those with mild disease when compared to the other disease severity groups or the uninfected (*p* = 0.86). Myristoleic (C14:1n5), eicosenoic (C20:1n9), and palmitoleic acids (C16:1n7) were not significantly different between the disease severity groups or compared to the uninfected adults (Table 2).

### Polyunsaturated fatty acids

The levels of seven polyunsaturated fatty acids were quantified and analyzed for significant differences between the disease severity groups and uninfected individuals (Table 2). Figure 1E shows γ-linolenic acid (GLA) (C18:3n6) was significantly lower in the adults with moderate disease compared to the uninfected adults (9.1 vs 22.4 μg/ml, *p* = 0.03). Levels of this fatty acid were not significantly different in the mild and severe disease groups compared to other disease severities and the uninfected (*p* = 0.87 and *p* = 0.80, respectively). Additionally, linoleic acid (C18:2n9) was significantly higher in adults with mild disease (527.9 μg/ml) and uninfected adults (536.2 μg/ml) when compared to the adults with moderate disease (305.8 μg/ml) (*p* = 0.04 and *p* = 0.03, respectively) (Figure 1F). Eicosapentaenoic acid (C20:5n3) (EPA) levels were significantly lower in the mild group (11.4 μg/ml) and the moderate group (6.6 μg/ml) when compared to the uninfected adults (22.4 μg/ml) (*p* = 0.03 and *p* = 0.007, respectively) (Figure 1G). Docahexaenoic (C22:6n3), arachidonic (C20:4n6), and linolenic (C18:3n3) acid levels were detectable, though levels of these fatty acids were not significantly different between the disease severity groups or uninfected adults. Docosadienoic acid (C22:2n6) was not detected in any sample. Supplementary Table 1 shows all quantified PFA for the entire cohort.

### Post-acute sequelae of COVID-19 associated fatty acid changes

Twenty-three participants completed two additional study visits several weeks after their initial infection (26–229 days post-PCR + diagnosis), for a total of three study visits. Twelve participants developed PASC (sampled at 55.8 ± 20.8 days post-PCR + diagnosis), while the remaining eleven participants did not report experiencing persisting symptoms when sampled at 121.2 ± 61.1 days post-PCR + diagnosis. Fatty acid levels were compared between study visits within the PASC vs No PASC groups to assess for fluctuations in fatty acids based on days post PCR +. There was variation in the study visits due to days post PCR +. Individuals who experienced PASC demonstrated significantly lower levels of GLA during the early recovery stage (16.8 ± 13.8 days post-PCR +) compared to samples taken during later recovery stages (55.8 ± 20.8 days post-PCR +) (20.4 vs 35.7 μg/ml) (*p* = 0.02). At 113.5 ± 23.4 days post-PCR +, mean levels of GLA were higher than levels observed at 16.8 ± 13.8 days post-PCR + (28.0 vs 20.4 μg/ml) but were lower than those observed at 55.8 ± 20.8 days post-PCR + (28.0 vs 35.7 μg/ml). This difference between 55.8 ± 20.8 days and 113.5 ± 23.4 days post-PCR + was not statistically significant, though it is interesting to note that GLA levels decreased several months into the recovery period for individuals who experience PASC. Levels of GLA between the acute and convalescent stages of recovery were not statistically different in those who did not develop PASC, and levels remained relatively stable during all periods of infection (Table 3). Similar to the findings observed for GLA, Table 3 illustrates that levels of EPA were significantly lower at 16.8 ± 13.8 days post-PCR + compared to 55.8 ± 20.8 days post-PCR + (11.1 vs 24.8 μg/ml, *p* = 0.0003) for those who developed PASC. Samples taken at 113.5 ± 23.4 days post-PCR + showed higher levels of EPA compared to 16.8 ± 13.8 days post-PCR + (14.2 vs 11.1 μg/ml), though these levels were lower than those observed at 55.8 ± 20.8 days post-PCR + (14.2 vs 24.8 μg/ml). This difference between 113.5 ± 23.4 days post-PCR + and 16.8 ± 13.8 days post-PCR + was not statistically significant, though it is notable that similar fluctuations in EPA levels were not observed in the No PASC group (Table 3). BMI as a covariate was not significantly related to the fatty acid changes.

**TABLE 3:** Lower levels of anti-inflammatory fatty acids during early stages of recovery in adults with post-acute sequelae of COVID-19 (PASC).

	No PASC (*N* = 12)					PASC (*N* = 11)				
	Days post PCR +			*p*-value		Days post PCR +			*p*-value	
μg/ml	66.2 ± 54.6 V1	121.2 ± 61.1 V2	187.8 ± 69.1 V3	V1 V2	V1 V3	16.8 ± 13.8 V1	55.8 ± 20.8 V2	113.5 ± 23.4 V3	V1 V2	V1 V3
**Saturated fatty acids** (**μg/ml)**										
Behenic (C22)	2.7 ± 1.1	2.78 ± 1.4	3.33 ± 1.7	1.00	0.87	1.9 ± 0.73	2.8 ± 1.56	2.5 ± 2.2	0.24	0.95
Palmitic (C16)	424.5 ± 214.2	530.1 ± 440.0	487.1 ± 334.4	0.95	0.99	424.5 ± 214.2	787.3 ± 342.4	612.3 ± 291.9	0.38	0.96
Lignoceric (C23)	2.7 ± 1.1	2.7 ± 1.45	3.2 ± 1.6	0.99	0.95	1.8 ± 0.78	2.6 ± 1.5	2.3 ± 1.9	0.20	0.92
Lauric (C12)	3.3 ± 3.34	4.4 ± 5.40	3.31 ± 3.1	0.99	1.00	2.27 ± 1.1	5.86 ± 3.8	6.4 ± 5.8	0.21	0.26
Stearic (C18)	179.0 ± 67.2	201.0 ± 96.6	193.4 ± 86.8	0.99	0.99	163.0 ± 54.7	240.6 ± 109.2	192.9 ± 72.9	0.06	0.76
Pentadecaoic (C15)	4.2 ± 3.1	4.9 ± 4.8	4.7 ± 3.8	0.99	0.99	4.16 ± 2.4	6.71 ± 2.8	4.8 ± 1.9	0.10	0.87
Arachidic (C20)	1.8 ± 0.34	1.5 ± 0.80	1.8 ± 0.90	0.99	0.53	1.13 ± 0.44	1.82 ± 1.0	1.3 ± 0.7	0.06	0.87
**Monounsaturated fatty acids (μg/ml)**										
Oleic (C18:1n9)	127.3 ± 58.4	161.9 ± 143.0	146.9 ± 97.4	0.95	0.99	166.5 ± 50.1	224.8 ± 88.3	170.6 ± 60.9	0.38	1.00
Myristoleic (C14:1n5)	0.7 ± 0.26	1.02 ± 1.1	0.94 ± 1.0	0.98	0.99	1.0 ± 0.75	1.93 ± 1.7	0.7 ± 0.4	0.30	0.99
Eicosenoic (C20:1n9)	1.84 ± 1.1	2.27 ± 3.1	2.70 ± 2.4	0.97	0.92	2.9 ± 1.2	3.9 ± 2.24	2.5 ± 1.3	0.71	0.95
Palmitoleic (C16:1n7)	37.2 ± 29.8	46.5 ± 59.0	44.2 ± 45.0	1.00	0.99	45.8 ± 18.5	76.3 ± 41.5	57.9 ± 26.0	0.19	0.83
**Polyunsaturated fatty acids (μg/ml)**										
γ-linolenic (C18:3n6)	25.2 ± 13.1	26.3 + 17.1	29.8 ± 23.4	1.00	0.99	20.4 + 18.7	35.7 + 21.6	28.0 ± 14.2	**0.02**	0.08
Eicosapentaenoic (C20:5n3)	19.1 + 12.2	17.0 + 8.7	20.0 ± 9.5	0.99	0.99	11.1 + 8.0	24.8 + 13.4	14.2 ± 8.5	**0.0003**	0.42
Docosahexaenoic (C22:6n3)	46.8 ± 25.7	47.9 ± 19.3	49.9 ± 19.7	0.99	0.91	40.9 ± 11.7	52.5 ± 25.5	44.3 ± 20.9	0.63	1.00
Linoleic (C18:2n9)	508.0 ± 214.7	575.6 ± 205.9	539.7 ± 224.9	0.86	0.99	448.8 ± 179.9	617.3 ± 237.8	527.6 ± 271.3	0.09	0.80
Linolenic (C18:3n3)	15.9 ± 7.4	21.3 ± 21.9	19.5 ± 12.6	0.99	0.99	20.8 ± 13.6	32.4 ± 19.5	20.2 ± 10.4	0.26	1.00
Arachidonic (C20:4n6)	220.4 ± 83.3	234.8 ± 101.6	249.1 ± 100.7	0.99	0.72	189. ± 62.6	237. ± 80.1	225.0 ± 97.6	0.11	0.49

Values presented as mean ± standard deviation, apart from days post PCR, which are presented as mean ± average deviation. Abbreviations V1, V2, and V3 are study visits 1, 2, and 3, respectively. Linear mixed model was performed to compare the fatty acids quantification for no PASC and PASC groups at T2 and T3 compared to their T1. SAS v9.4 (SAS Institute Inc., Cary, NC) was used to perform all statistical analyses. P < 0.05 significant; bold = significant.

## Discussion

In this pilot study of forty-one COVID-19 survivors and nine adults without history of infection, there were clear differences in the plasma fatty acid (PFA) profiles of those who experienced a moderate or severe disease course compared to the adults with mild disease and to the uninfected. Individuals who developed PASC also showed differences in PFA levels compared to the adults who did not develop PASC. Among the saturated fatty acid profiles, levels of palmitic acid were significantly higher in those who experienced a severe disease course relative to the uninfected adults. Previous analysis of fatty acid metabolism in COVID-19 has revealed that palmitic acid is likely to play a role in viral entry to host cells, as palmitic acid is known to lipidate the cysteine residues found on the SARS-CoV-2 spike and envelope proteins (26). Thus, individuals with higher levels of this fatty acid may have been more susceptible to viral invasion and subsequently developed a more severe disease course. Additionally, SARS-CoV-2 has also been hypothesized to promote activation of palmitic acid synthesis via upregulation of the genes responsible for signaling the transcription of fatty acid synthase (FASN), acetyl-CoA carboxylase (ACC), and stearoyl-CoA desaturase 1 (SCD1) (26). In doing so, the virus increases the lipid stock and further promotes its replication, increasing viral load within the body (26). Among the other saturated fatty acids included in this study, behenic and lignoceric acid levels were significantly different in adults with moderate disease compared to the uninfected individuals, with both showing depletion in the infected adults. Depletion of behenic acid in COVID-19 patients has been observed in one other study and is thought to be correlated with adverse disease outcomes, including intestinal inflammation and altered serum metabolites (27). Although levels of lignoceric acid in COVID-19 patients have not previously been explored, depletion in this fatty acid has been linked to unfavorable immune responses, particularly in cases of autoimmune diseases and response to cancer treatments (28, 29). Additional reports have also linked higher levels of lignoceric acid to decreased incidence of age-related diseases, thus further implicating its potential protective role within the body (30).

Analysis of unsaturated fatty acid profiles within this cohort of COVID-19 survivors revealed several significant findings. Oleic acid was significantly higher in those who experienced a severe disease course compared to the adults with moderate disease. Individuals within the moderate and severe disease groups were of similar BMI, thus indicating that this difference was independent of obesity level. Elevation of oleic acid in COVID-19 was previously described by Barberis et al., who showed that levels of oleic acid directly correlated with disease severity (31). Interestingly, unsaturated fatty acids, such as oleic, arachidonic, and linoleic acid, have been shown to mediate antiviral activity by disintegrating the envelope of certain animal viruses, including herpes and influenza (32). Oleic acid may not demonstrate this same capacity with the envelope of SARS-CoV-2 given its direct correlation with disease severity. However, depletion in linoleic acid was observed in the adults with moderate disease compared to the mild and uninfected adults, and this may be attributed to the capacity for linoleic acid to interfere with the envelope structure of SARS-CoV-2. In fact, linoleic acid has been shown to play a structural role in preventing entry of the SARS-CoV-2 virus into host cells by binding the spike protein on the viral envelope and locking it in a conformation that inhibits interaction with ACE2 (33). Additional studies have also implicated that linoleic acid, along with linolenic (n3) and EPA, interfere with the receptor binding domain sequence of the SARS-CoV-2 virus, further blocking interacting with host ACE2 receptors (34). While linolenic (n3) levels were not different between the disease severity groups in this study, GLA (n6) levels were significantly lower in the adults with moderate disease compared to the uninfected. Similar to the effect of linoleic acid previously described, omega-3 fatty acids, such as EPA and DHA, have been found to interfere with SARS-CoV-2 spike protein conformation to prevent interaction with host cells and indeed, EPA levels were significantly lower in the adults with moderate and mild disease compared to the uninfected (35). These findings support the postulations of Baral et al. and Mazidimoradi et al. in demonstrating that polyunsaturated fatty acids may act as important mediators in determining COVID-19 disease severity (36, 37).

In addition to analyzing the differences in fatty acid levels between individuals based on disease severity, this study compared the relative levels of individual fatty acids between three study visits to determine if depletion was associated with the development of PASC. To our knowledge, this is the first study to explore such a relationship. Currently, reference ranges for individual fatty acids are poorly defined and are typically represented as a percentage of total PFA composition, though a small number of population studies have defined the reference ranges of a limited suite of fatty acids in healthy adults (38, 39). EPA is typically found in the range of 12.0–68.6 μmol/L, while GLA concentrations are found in the range of 9.7–37.3 μmol/L (38). For the purposes of this study, depletion was measured based on significantly lower levels of fatty acid concentrations when measured during the early recovery period (16.8 ± 13.8 days post-PCR +) versus later recovery stages (55.8 ± 20.8 and 113.5 ± 23.4 days post-PCR +). Of the fatty acids measured, EPA levels were significantly lower at 16.8 ± 13.8 days post-PCR + compared to 55.8 ± 20.8 days post-PCR + in the individuals who developed PASC but were not different between the visits in the adults who did not experience persistent symptoms. This same relationship was observed for GLA, with levels in the PASC group being significantly lower at 16.8 ± 13.8 days post-PCR + compared to 55.8 ± 20.8 days post-PCR +. While the levels of EPA and GLA at 113.5 ± 23.4 days post-PCR + were not significantly different from those measured at 16.8 ± 13.8 days post-PCR + and 55.8 ± 20.8 days post-PCR +, the mean levels of these fatty acids did decrease at the 113.5 ± 23.4 day post-PCR + visit for those with PASC. These fluctuations in EPA and GLA levels were not observed in the individuals who did not develop PASC. It should be noted that the collection times (days post PCR +) differed between the PASC and No PASC groups, with sample collections occurring closer to initial PCR + in the PASC group (study V1 occurred at 16.8 ± 13.8 days post PCR + in the PASC group vs 66.2 ± 54.6 days post PCR + for No PASC). However, when comparing similar days post PCR + between groups (i.e., 55.8 ± 20.8 in PASC group vs 66.2 ± 54.6 in No PASC group and 113.5 ± 23.4 in PASC group vs 121.2 ± 61.1 in No PASC group), the symptoms reported by individuals experiencing PASC remained consistent. In other words, at all study visits, individuals with PASC continued to experience persisting symptoms, while those in the No PASC group remained asymptomatic. Thus, despite the inconsistency in days post PCR + between the groups, it appears that the fluctuations in GLA and EPA did correlate to the persistence of symptoms. Additionally, it is notable that there was more fluctuation in EPA and GLA levels between each study visit in the PASC group, while levels of EPA and GLA remained fairly consistent in the No PASC group (see Table 3).

Although further research is warranted, these preliminary findings suggest that depletion in EPA and GLA may be a predictor for the development of persistent symptoms beyond the acute stage of infection. This correlation may be due to the anti-inflammatory properties of these fatty acids, as the development of PASC is increasingly linked to persistent elevations of inflammatory factors, such as C-reactive protein and interleukin-6 (IL-6) (40, 41). EPA has previously been shown to attenuate pro-inflammatory cytokines, and the benefits of supplementation to reduce COVID-19 severity has been suggested, with one small study indicating significantly improved survival rates after EPA supplementation in acutely ill patients (42–49). Although GLA is an omega 6 fatty acid, which are typically associated with pro-inflammatory effects, GLA has been found to demonstrate anti-inflammatory properties through its longer-chain derivative, 15-hydroxyeicosatraenoic acid, which acts to inhibit the formation of the pro-inflammatory leukotriene B4 (LTB4) (50, 51). The LTB4 pathway appears to be upregulated in individuals with severe COVID-19, thus inhibition by GLA may help prevent adverse disease outcomes (52). Additional studies have also shown that GLA acts to mediate endothelial cell tumor necrosis factor alpha (TNF-α) expression and reduce production of IL-6, thereby further quelling inflammation (53). Interestingly, levels of arachidonic acid, a pro-inflammatory fatty acid, were not significantly different between the disease severity groups in this cohort and were not associated with the development of PASC. However, additional studies analyzing the PFA profiles of COVID-19-infected individuals showed arachidonic acid levels were elevated during acute infection, and this elevation was associated with severe disease (31, 54).

Preliminary findings from this cohort demonstrate significant differences in fatty acid profiles in COVID-19-infected adults with more significant disease outcomes compared to those with mild infection or no infection, thus demonstrating the potential for fatty acids to act as key modifiers in the disease course. Although the results of this study are promising, further study is needed given the limited size of this pilot cohort. Due to sample size limitations and the pilot nature of this study, the demographics of the infected individuals were not mirrored in the individuals comprising the uninfected group. As shown in Table 1, the mean BMI of the uninfected group was similar to the mean BMI of the mild disease group but was much lower than that of the moderate and severe disease groups, thus presenting a limitation in the comparison of the moderate and severe disease groups to the uninfected. There was also more racial and ethnic diversity in the moderate and severe disease groups compared to the uninfected, which further limited comparison. Future studies should incorporate more demographic diversity in the uninfected group to yield more accurate comparisons. Additional difficulties in accurate comparison included sample collection dates, as there was notable difference in the days post initial PCR +, particularly in the comparison between individuals with PASC versus those without PASC. At the time of analysis, few participants in the NoCO CoBIO cohort had completed three study visits, thus presenting challenges in controlling for the amount of time that had passed between collection dates and initial PCR +. Future studies should control for this variable to better define the relationship between fatty acid profiles and PASC development. The use of convalescent plasma in many of the participants during early stages of the pandemic presented challenges in the analysis of fatty acid profiles obtained during the acute stage of infection, particularly among those with severe disease. This study did include patients that did not receive convalescent plasma and trends for differential profiles remained apparent. Finally, the precise role of diet to induce PFA changes is not definitive given *de novo* lipogenesis also accounts for the composition of PFA, particularly palmitic, stearic, palmitoleic, oleic, and myristoleic acid (55). However, the endogenous synthesis of polyunsaturated fatty acids, particularly EPA and DHA is limited, with some studies suggesting as little as 2–10% of *de novo* lipogenesis of these fatty acids occurs (56, 57). The findings of this study suggest that long-chain fatty acids are associated with lower incidence of PASC, thus making dietary intake of EPA and GLA a promising and safely administered applications for the convalescent stages of disease and for those at risk for PASC.

Further investigation into the role of PFA in determining COVID-19 disease severity and the development of PASC is warranted in larger cohorts based on findings of this pilot observational study indicating individuals with more severe COVID-19 infections have altered plasma fatty acid profiles. As potent inflammatory mediators and structural modifiers of SARS-CoV-2, dietary supplementation of behenic, lignoceric, linoleic, GLA, and EPA may be a cost effective and non-invasive method of preventing or controlling PASC, and particularly in overweight or obese individuals who are of heightened risk of severe disease outcomes.

## Data availability statement

The original contributions presented in this study are included in the article/Supplementary material, further inquiries can be directed to the corresponding author.

## Ethics statement

The studies involving human participants were reviewed and approved by Colorado State University Research Integrity and Compliance Review Office Institutional Review Board [IRB; protocol 2105 (20-10063H)] and University of Colorado Health Institutional Review Board (Colorado Multiple IRB 20-6043). The patients/participants provided their written informed consent to participate in this study.

## Author contributions

ER and JD conceived, designed and conducted the research, and performed funding acquisition. SS and BB completed co-writing-original draft preparation. SS, BB, SL, EG, TW, TD, KM, MH-T, GE, and ER reviewed and edited the manuscript. SR performed the formal analysis. GD performed plasma fatty acid determinations. BB, SS, SL, KB, MT, JH, and NN administered symptom survey and data entry for analysis. All authors have read and agreed to the published version of the manuscript.
